# Supplementary material for: P-TRAP: a Panicle Trait Phenotyping tool
Source: BMC Plant Biol. 2013 Aug 29;13:122. doi: 10.1186/1471-2229-13-122 (PMC3848748; doi:10.1186/1471-2229-13-122)
Supplement: Additional file 7 — P-TRAP source code. The Netbeans project that contains the source code of the application. [file 1471-2229-13-122-S7.zip › javadoc.zip!/javadoc/org-basmati-workspace/org/basmati/workspace/ResultProducer.html]

ResultProducer (WorkSpace)


JavaScript is disabled on your browser.


- Package
- Class
- Tree
- Deprecated
- Index
- Help

*org.basmati.workspace 1.0*

- Prev Class
- Next Class

- Frames
- No Frames

- All Classes

- Summary:
- Nested |
- Field |
- Constr |
- Method

- Detail:
- Field |
- Constr |
- Method


org.basmati.workspace

## Class ResultProducer

- java.lang.Object
- - org.basmati.workspace.ResultProducer

- ---

    

  ```
  public abstract class ResultProducer
  extends java.lang.Object
  ```

  It saver of all result files.

  Author:
  :   faroq

- - ### Constructor Summary

    Constructors

    Constructor and Description

    `ResultProducer()`
  - ### Method Summary

    Methods

    | Modifier and Type | Method and Description |
    | --- | --- |
    | `static boolean` | `createFile(org.bioalg.graphprocessor.GraphModel model, java.lang.String imagefile, java.io.File resutlfile)` Given memory objects, this function writes a single result xml file that holds all information |
    | `static boolean` | `createFile(org.bioalg.graphprocessor.GraphModel model, java.lang.String imagefile, java.lang.String resutlfilename, java.lang.String resultpath)` Given memory objects, this function writes a single result xml file that holds all information |
    | `static boolean` | `createFile(org.biolag.particleprocessor.ParticlesModel model, java.lang.String imagefile, java.io.File resutlfile)` Write down the input particle model to an XML file. |
    | `static boolean` | `createFile(org.biolag.particleprocessor.ParticlesModel model, java.lang.String imagefile, java.lang.String resutlfilename, java.lang.String resultpath)` Write down the input particle model to an XML file. |

    - ### Methods inherited from class java.lang.Object

      `clone, equals, finalize, getClass, hashCode, notify, notifyAll, toString, wait, wait, wait`

- - ### Constructor Detail


    - #### ResultProducer

      ```
      public ResultProducer()
      ```
  - ### Method Detail


    - #### createFile

      ```
      public static boolean createFile(org.bioalg.graphprocessor.GraphModel model,
                       java.lang.String imagefile,
                       java.lang.String resutlfilename,
                       java.lang.String resultpath)
      ```

      Given memory objects, this function writes a single result xml file that
      holds all information

      Parameters:
      :   `model` - A graph model that represents the image structure
      :   `imagefile` - the source image file
      :   `resutlfilename` - the result file
      :   `resultpath` - the result path, where results have to be stored in the
          project

      Returns:
      :   true if success or false otherwise


    - #### createFile

      ```
      public static boolean createFile(org.bioalg.graphprocessor.GraphModel model,
                       java.lang.String imagefile,
                       java.io.File resutlfile)
      ```

      Given memory objects, this function writes a single result xml file that
      holds all information

      Parameters:
      :   `model` - graph model
      :   `imagefile` - the image file path
      :   `resutlfile` - a File object for the which the result file has to be
          save

      Returns:


    - #### createFile

      ```
      public static boolean createFile(org.biolag.particleprocessor.ParticlesModel model,
                       java.lang.String imagefile,
                       java.lang.String resutlfilename,
                       java.lang.String resultpath)
      ```

      Write down the input particle model to an XML file.

      Parameters:
      :   `model` - `ParticlesModel`
      :   `imagefile` - The name of the source image
      :   `resutlfilename` - The name of the result file
      :   `resultpath` - the result path

      Returns:
      :   true if success, false otherwise.


    - #### createFile

      ```
      public static boolean createFile(org.biolag.particleprocessor.ParticlesModel model,
                       java.lang.String imagefile,
                       java.io.File resutlfile)
      ```

      Write down the input particle model to an XML file.

      Parameters:
      :   `model` - particles model
      :   `imagefile` - the image file
      :   `resutlfile` - the result file

      Returns:
      :   true if success false otherwise


- Package
- Class
- Tree
- Deprecated
- Index
- Help

*org.basmati.workspace 1.0*

- Prev Class
- Next Class

- Frames
- No Frames

- All Classes

- Summary:
- Nested |
- Field |
- Constr |
- Method

- Detail:
- Field |
- Constr |
- Method
